# Interleukin-Driven Inflammation and Cardiovascular Risk in People Living with HIV: Mechanisms, Biomarkers, and Emerging Preventive Strategies

**DOI:** 10.3390/medsci14060629

**Published:** 2026-10-01

**Authors:** Asbiel Felipe Garibaldi-Ríos, Martha Patricia Gallegos-Arreola, Jesús Magdiel García-Díaz, Belinda Claudia Gómez-Meda, Jazmín Márquez-Pedroza, Jhonathan Cárdenas-Bedoya, Juan Ernesto Gutiérrez-Sevilla, David Israel Javalera-Castro, Martha Escoto-Delgadillo, Antonio Kobayashi-Gutiérrez, María de Lourdes Lemus-Varela, Blanca Miriam Torres-Mendoza

**Affiliations:** 1 División de Genética, Centro de Investigación Biomédica de Occidente (CIBO), Centro Médico Nacional de Occidente (CMNO), Instituto Mexicano del Seguro Social (IMSS), Guadalajara 44340, Jalisco, Mexico; 2 Unidad de Biotecnología Médica y Farmacéutica, Centro de Investigación y Asistencia en Tecnología y Diseño del Estado de Jalisco A.C. (CIATEJ), Av. Normalistas 800, Col. Colinas de la Normal, Guadalajara 44270, Jalisco, Mexico; jegarcia_al@ciatej.edu.mx; 3 Instituto de Genética Humana “Dr. Enrique Corona Rivera”, Departamento de Biología Molecular y Genómica, Centro Universitario de Ciencias de la Salud (CUCS), Universidad de Guadalajara (UdeG), Guadalajara 44340, Jalisco, Mexico; 4 Laboratorio de Inmunodeficiencias y Retrovirus Humanos, División de Neurociencias, Centro de Investigación Biomédica de Occidente (CIBO), Centro Médico Nacional de Occidente (CMNO), Instituto Mexicano del Seguro Social (IMSS), Guadalajara 44340, Jalisco, Mexicodrkoba@hotmail.com (A.K.-G.); 5 Departamento de Disciplinas Filosófico Metodológicas e Instrumentales, Centro Universitario de Ciencias de la Salud (CUCS), Universidad de Guadalajara (UdeG), Guadalajara 44340, Jalisco, Mexico; 6 División de Medicina Molecular, Centro de Investigación Biomédica de Occidente (CIBO), Centro Médico Nacional de Occidente (CMNO), Instituto Mexicano del Seguro Social (IMSS), Guadalajara 44340, Jalisco, Mexicolulalemus@gmail.com (M.d.L.L.-V.); 7 Centro Universitario de Ciencias Biológicas y Agropecuarias (CUCBA), Universidad de Guadalajara (UdeG), Guadalajara 44600, Jalisco, Mexico

**Keywords:** HIV, interleukins, IL-6, IL-18, cardiovascular risk, chronic inflammation, atherosclerosis, antiretroviral therapy, inflammatory biomarkers, monocyte activation, microbial translocation, endothelial dysfunction

## Abstract

Heart and vascular complications now rank among the leading health threats facing individuals with HIV, a reality that continues even when modern drug regimens successfully silence viral replication. Sustained immune system overdrive and persistent low-grade systemic inflammation appear central to this heightened vulnerability. This narrative review integrates current understanding of how inflammatory signaling molecules, particularly interleukins, connect to heightened cardiovascular danger in HIV-positive populations, while exploring mechanistic underpinnings, cohort-derived evidence, and prospective treatment possibilities. Elevated concentrations of proinflammatory messengers such as IL-6 and IL-18, alongside indicators of myeloid cell activation including sCD14 and sCD163, characterize the HIV-positive state and independently forecast arterial disease progression, clinical cardiac events, and shortened survival. Notably, this inflammatory signature endures despite pharmacologically induced viral quiescence. Disturbances in protective lipoprotein function, heightened clotting propensity, and leakage of gut-derived bacterial components into the bloodstream each amplify vascular injury. Inflammatory cytokines occupy a central position in HIV-associated cardiovascular pathobiology, serving dual roles as disease predictors and candidate treatment targets. Importantly, recent evidence from the REPRIEVE randomized clinical trial demonstrated that pitavastatin therapy reduced major adverse cardiovascular events by approximately 35% in people living with HIV receiving antiretroviral therapy, highlighting inflammation-modulating strategies as a new foundation of cardiovascular prevention in this population.

## 1. Introduction

Vascular and cardiac pathology has risen to prominence as a clinical priority for healthcare providers managing HIV-positive patients in the current therapeutic era. While combination drug regimens have fundamentally altered disease trajectories, converting what was once a universally fatal condition into a manageable lifelong state, the specter of premature cardiovascular morbidity remains [1,2]. Epidemiological surveillance consistently reveals that affected individuals experience approximately 1.5–2-fold higher rates of major cardiovascular events, including coronary or cerebrovascular events, compared with uninfected counterparts sharing similar demographic profiles [3,4].

The multinational D:A:D surveillance study (Data Collection on Adverse Events of Anti-HIV Drugs), which tracks cardiovascular outcomes in treated individuals, generated some of the earliest quantitative evidence linking cumulative antiretroviral exposure to rising rates of myocardial infarction [5]. Subsequent analytical work and contemporary scientific statements, however, clarified that traditional vascular risk determinants, ongoing systemic inflammation, and virus-specific effects carry comparable pathogenic weight [4,6].

A watershed moment arrived with results from the SMART investigation, an interventional trial originally conceived to test treatment-sparing approaches. Contrary to expectations, participants randomized to CD4-guided intermittent therapy had a higher rate of major cardiovascular, renal or hepatic disease than continuously treated counterparts (hazard ratio 1.7; 95% CI 1.1–2.5) [7,8]. This observation fundamentally redirected scientific attention toward immunological and inflammatory processes as drivers of vascular damage. Ancillary biomarker analyses from classic cohorts further revealed that circulating IL-6 and D-dimer concentrations each independently forecast mortality risk [9]. Table 1 profiles the major prospective investigations informing current understanding of inflammation-cardiovascular linkages in this population.

When contemporary cohorts benchmarked treated enrollees against participants in large cardiovascular epidemiology studies, they observed that successfully treated patients still carried significantly higher circulating levels of acute-phase reactants, IL-6, and coagulation markers than their uninfected peers [18,19]. Parallel observations throughout geographically and demographically varied populations have since cemented residual inflammation as a hallmark biological feature of treated chronic infection [20].

This review undertakes an extensive examination of published evidence connecting interleukin biology to cardiovascular vulnerability in HIV-positive individuals. We dissect the physiological pathways through which these signaling molecules accelerate arterial disease, summarize observational findings from landmark international cohorts, evaluate how different antiretroviral strategies modulate inflammatory tone, and consider emerging intervention approaches targeting persistent immune activation.

## 2. Materials and Methods

### 2.1. Search Strategy

This article is a narrative review. The literature discussed was identified through searches of PubMed/MEDLINE and Scopus carried out on 15 September 2026. Searches were limited to publications in English issued between January 1996, when combination antiretroviral therapy became widely available, and September 2026.

The following string was used in PubMed/MEDLINE: (“HIV”[MeSH Terms] OR “HIV Infections”[MeSH Terms] OR “HIV”[tiab] OR “people living with HIV”[tiab] OR “PLWH”[tiab]) AND (“Interleukins”[MeSH Terms] OR “interleukin”[tiab] OR “IL-6”[tiab] OR “IL-18”[tiab] OR “IL-1beta”[tiab] OR “tumor necrosis factor”[tiab] OR “inflammation”[MeSH Terms] OR “immune activation”[tiab] OR “microbial translocation”[tiab] OR “sCD14”[tiab] OR “sCD163”[tiab] OR “lipoproteins, HDL”[MeSH Terms] OR “cholesterol efflux”[tiab]) AND (“Cardiovascular Diseases”[MeSH Terms] OR “Atherosclerosis”[MeSH Terms] OR “cardiovascular risk”[tiab] OR “myocardial infarction”[tiab] OR “carotid intima-media thickness”[tiab] OR “endothelial dysfunction”[tiab] OR “coronary plaque”[tiab]).

The equivalent string in Scopus was: TITLE-ABS-KEY((“HIV” OR “human immunodeficiency virus” OR “people living with HIV” OR “PLWH”) AND (“interleukin” OR “IL-6” OR “IL-18” OR “IL-1beta” OR “tumor necrosis factor” OR “inflammation” OR “immune activation” OR “microbial translocation” OR “sCD14” OR “sCD163” OR “high-density lipoprotein” OR “cholesterol efflux”) AND (“cardiovascular” OR “atherosclerosis” OR “myocardial infarction” OR “intima-media thickness” OR “endothelial dysfunction” OR “coronary plaque”)) AND PUBYEAR > 1995 AND LANGUAGE(english).

### 2.2. Evidence Selection

Studies were selected by the authors on the basis of topical relevance. Priority was given to long-term observational cohorts and randomized trials from established research networks, including SMART, VACS, MACS, WIHS, D:A:D, and REPRIEVE, to cross-sectional analyses comparing virologically suppressed individuals with HIV-negative controls, and to the mechanistic in vitro and animal studies needed to explain the pathways under discussion. Publications were considered only if they reported cardiovascular or inflammatory outcomes and were available as full-text articles. Experimental and general-population studies falling outside the scope of the searches described above were identified from the reference lists of retrieved articles.

The review addresses five domains. These are interleukin biology and signaling, the epidemiological associations between inflammatory biomarkers and cardiovascular outcomes in HIV cohorts, monocyte activation and microbial translocation, high-density lipoprotein dysfunction, and the inflammatory effects of antiretroviral therapy and of emerging preventive interventions. For each principal study, the design, sample size, enrollment period, follow-up duration and biomarkers assessed are summarized in Table 1, and the type and population of each source are indicated in the corresponding tables throughout the review.

## 3. Interleukin 6 and Cardiovascular Risk

### 3.1. Biological Characteristics and Signaling

Among inflammatory mediators implicated in cardiovascular pathology, IL-6 stands out for its capacity to simultaneously orchestrate both acute defensive responses and chronic tissue-damaging processes. This molecule participates in coordinating early innate immunity and shaping longer-term adaptive reactions, a functional duality that Rose-John and colleagues have emphasized in the recent literature [21]. Cellular sources are remarkably diverse, encompassing circulating immune cells, vascular wall constituents, connective tissue elements, and metabolically active adipose depots [21,22,23].

Signal transduction proceeds through two fundamentally different routes with distinct biological consequences. One pathway requires direct cytokine engagement with membrane-tethered receptors concentrated on hepatocytes and selected leukocyte populations. An alternative mechanism, termed trans-signaling, dramatically expands the molecule’s reach: circulating soluble receptor variants capture the cytokine and deliver it to virtually any tissue expressing the ubiquitous gp130 signal transducer [24,25]. This latter pathway bears responsibility for chronic proinflammatory and vessel-damaging effects.

### 3.2. Epidemiological Evidence from HIV Cohorts

Pivotal observations emerged from banked plasma specimens in the SMART trial, where higher IL-6 and D-dimer concentrations predicted all-cause mortality independently of CD4 count and HIV RNA level [9]. When the same specimens were set against the general-population MESA and CARDIA cohorts, patients on antiretroviral therapy with suppressed viremia still showed higher interleukin-6, high-sensitivity C-reactive protein, D-dimer, and cystatin C [26]. Smaller cross-sectional work extended the pattern to macrophage and endothelial activation, where raised soluble CD163 and soluble VCAM-1 were accompanied by a reciprocal fall in soluble TWEAK, together with higher fibrinogen [27,28,29]. Table 2 compares these concentrations between suppressed and uninfected populations.

The Veterans Aging Cohort Study (VACS), whose design and enrollment Justice and colleagues set out in detail [30], allowed the same comparison on a far larger scale, drawing on a Biomarker Cohort of 1525 infected and 843 uninfected veterans [10,11]. Taken as a whole, HIV serostatus did not raise the prevalence of elevated IL-6, D-dimer, or sCD14 above that of uninfected veterans; the difference surfaced only after stratification by virological and immunological parameters [11]. Both detectable viremia (HIV-RNA ≥ 500 copies/mL) and severe depletion (CD4 < 200 cells/µL) carried substantially higher probabilities of an elevated marker, most clearly for IL-6 (odds ratio 2.25 at CD4 < 200 cells/µL) and for the monocyte-activation marker sCD14 (odds ratio 2.60 at the same threshold), underscoring how advancing immune compromise amplifies inflammatory signaling [11]. Table 3 sets out these subgroup estimates.

These same mediators carry clear prognostic weight. Within the SMART trial, participants in the highest quartile of IL-6 or D-dimer had unadjusted odds of death approximately 8-fold and 12-fold higher than those in the lowest quartile [9], while raised sCD14 independently predicted an approximately sixfold excess in mortality [31]; in VACS, D-dimer and sCD14 sharpened the prediction of death beyond a validated clinical index [32].

Evidence from the REPRIEVE trial population has since quantified how much of the residual risk these mediators account for. Among participants with suppressed infection and low-to-moderate predicted risk, IL-6 emerged as the biomarker most strongly tied to incident cardiovascular events, and the fraction of events attributable to it reached 18.6% [17]. That inflammatory markers behaved independently of lipid and cardiac markers in the same population argues that they index a pathway not addressed by lipid-lowering alone.

Recent examinations of cohort records identified elevated acute-phase reactant levels as independently associated with heart attack occurrence among HIV-positive patients after accounting for conventional vascular risk determinants [33]. Furthermore, high-resolution imaging evidence, including positron emission tomography, has revealed increased glucose uptake, a surrogate marker of metabolic inflammation, within the arterial walls of infected vs. uninfected subjects [18]. Serial imaging studies have demonstrated that carotid artery wall thickening progresses more rapidly in seropositive than in seronegative individuals, independent of traditional risk factor burden [34,35].

### 3.3. Mechanisms Promoting Arterial Disease

Multiple linked pathways explain how this cytokine accelerates vascular pathology. Serving as the principal driver of hepatic acute-phase responses, IL-6 upregulates production of CRP, clotting factors, and serum amyloid components, each independently linked to thrombotic and inflammatory vascular injury [36,37]. Recent population-based epidemiology established that circulating inflammatory markers predict cardiac events regardless of conventional risk profiles [38]. Table 4 catalogs the proatherogenic actions of major cytokines relevant to HIV. The mechanisms summarized below draw on two distinct bodies of evidence: work conducted in people living with HIV, and experimental or general-population studies from which these pathways are inferred to operate in the HIV setting. The type and population of each source are indicated in the table.

At the vessel wall level, IL-6 activates endothelial cells, triggering the display of adhesion receptors that capture circulating monocytes and facilitate their entry into subendothelial spaces [21,23,46]. Circulating levels of key adhesion molecules (sICAM-1, sVCAM-1, and E-selectin) independently predict adverse outcomes in patients with established coronary artery disease [47]. These molecular pathways correlate with arterial wall thickening and atherogenesis, as detailed in mechanistic reviews [48,49].

## 4. IL-18, Additional Cytokines, and Viral Coinfection

### 4.1. IL-18 Biology in HIV Infection

Initially defined by its potent interferon-gamma-inducing properties, IL-18 belongs to the IL-1 superfamily and exerts strong proinflammatory effects. Unlike many cytokines that require de novo transcription upon stimulation, cells constitutively store an inactive precursor form that requires inflammasome-mediated caspase-1 cleavage for activation [50,51]. This arrangement positions IL-18 as a sensitive readout of innate immune danger sensing and cellular stress.

Studies of HIV-positive populations consistently document elevated circulating IL-18, with particularly marked increases observed during uncontrolled viremia or concurrent hepatitis C virus (HCV) infection [52,53]. By powerfully stimulating interferon-gamma production, IL-18 promotes Th1-polarized inflammatory responses and drives macrophage activation toward phenotypes that accelerate atherogenesis [51].

### 4.2. Hepatitis C Coinfection

Dual infection with HCV identifies a subpopulation facing particularly elevated cardiovascular threat [54,55,56]. Supporting this concept, a systematic review by Osibogun and colleagues [57] reported that individuals with HIV/HCV coinfection exhibit a distinct proinflammatory and pro-atherogenic biomarker pattern relative to monoinfected or uninfected individuals. Specifically, this profile is characterized by increased levels of endothelial activation markers (such as sICAM-1 and sVCAM-1), elevated interleukin-6 and homocysteine concentrations, and greater carotid intima–media thickness, suggesting early vascular injury and endothelial dysfunction. Consistent with these biological alterations, Fernández-Montero and colleagues evaluated cardiovascular outcomes in a large cohort of HIV-infected individuals and found that HIV/HCV coinfection was independently associated with a higher incidence of cardiovascular events and related mortality compared with HIV monoinfection, even after adjusting for traditional cardiovascular risk factors [56].

## 5. Monocyte Activation and Gut Barrier Compromise

### 5.1. Soluble CD14 as a Prognostic Marker

CD14 functions as a pattern recognition coreceptor on innate immune cells, partnering with Toll-like receptor 4 to detect and clear bacterial endotoxin [58,59]. Landmark work by Sandler and colleagues demonstrated that elevated plasma sCD14 levels, a marker of monocyte activation driven by microbial translocation, were strongly associated with mortality in HIV infection, with individuals in the highest quartile showing approximately a sixfold higher risk of death compared with those in the lowest quartile [31]. This prognostic relationship remained intact after accounting for established predictors, positioning sCD14 among the most robust inflammatory markers of survival in this population. Supporting the role of innate immune activation in cardiovascular complications, Kelesidis et al. showed that higher levels of microbial translocation (LPS) and monocyte activation marker sCD14 were associated with accelerated progression of carotid intima–media thickness in people living with HIV [60].

### 5.2. Soluble CD163 and the TWEAK/Fn14 Axis

CD163 is a hemoglobin-haptoglobin scavenger receptor restricted to monocyte-macrophage lineage cells, with shedding occurring upon inflammatory activation [61]. Studies in untreated HIV infection have shown markedly elevated circulating sCD163 levels accompanied by reduced concentrations of soluble TWEAK [27]. To exert its biological functions, TWEAK binds to Fn14 (fibroblast growth factor-inducible 14), a receptor that is minimally expressed under normal conditions but strongly induced by tissue damage or inflammation. Once TWEAK engages with Fn14, it drives proinflammatory and profibrotic signaling, ultimately disrupting the normal equilibrium between inflammation and tissue repair [62]. Persistent macrophage activation has also been observed in individuals receiving suppressive antiretroviral therapy, with soluble markers such as CD14, CD163, and CD27 distinguishing people living with HIV from uninfected controls despite effective viral suppression [63]. In longitudinal analyses, elevated plasma sCD163 has been associated with the development of non-AIDS comorbidities, including chronic kidney, lung, and liver disease, supporting the concept that sustained monocyte–macrophage activation plays a role in long-term systemic complications of HIV infection [64]. Collectively, these findings show persistent monocyte–macrophage activation as a central component of chronic immune dysfunction in HIV, linking innate immune signaling with the development of multisystem comorbidities, including cardiovascular disease.

### 5.3. Intestinal Barrier Dysfunction and Bacterial Translocation

The gastrointestinal immune compartment experiences profound damage during HIV infection. Landmark work by Brenchley and colleagues demonstrated that CD4^+^ T-cell depletion occurs predominantly within mucosal tissues, particularly in the gastrointestinal tract, where CCR5-expressing CD4^+^ populations are preferentially lost across all stages of disease. This early and sustained mucosal immune disruption compromises epithelial barrier integrity and contributes to systemic immune activation [65].

**Figure 1:**
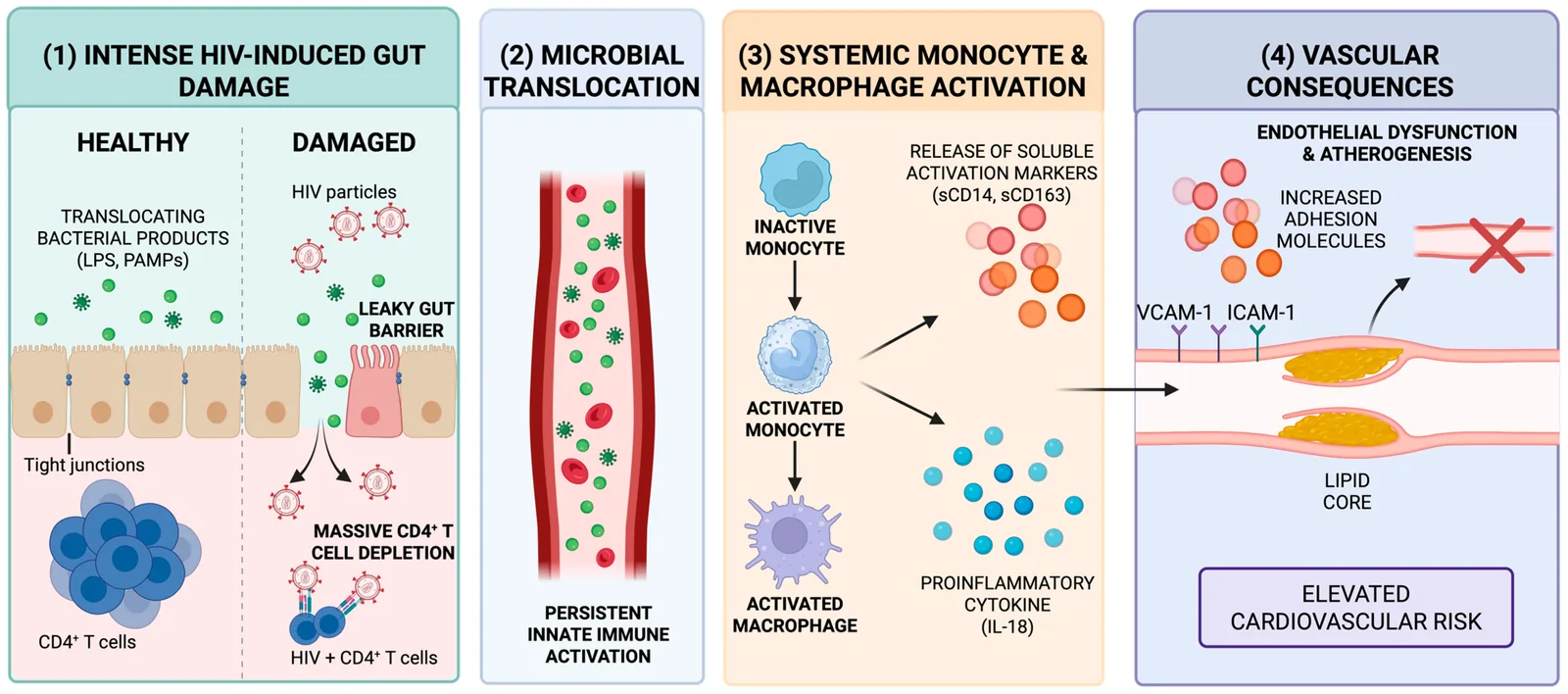
Gut–immunity–endothelium axis in cardiovascular risk associated with HIV. Schematic representation of the atherogenic cascade. (1) HIV infection induces damage to the intestinal mucosa and depletion of CD4+ T lymphocytes, leading to disruption of epithelial permeability. (2) This alteration allows the systemic translocation of bacterial products (LPS, PAMPs), which triggers (3) persistent activation of monocytes and macrophages, NLRP3 inflammasome activation, coupled with the discharge of proinflammatory cytokines such as IL-18 and IL-1β and soluble markers (sCD14, sCD163). (4) These mediators act on the vascular wall, promoting endothelial dysfunction by increasing the expression of adhesion molecules (VCAM-1, ICAM-1), thereby facilitating the development of atherosclerotic plaque and increasing cardiovascular risk. Created in BioRender. Garibaldi, A. (2026). https://BioRender.com/obzq8eq.

Vujkovic-Cvijin and colleagues showed that HIV infection is associated with gut microbial dysbiosis independent of sexual practices, defined by reduced microbial diversity and changes in key bacterial taxa. This altered microbiota profile correlates with inflammatory biomarkers and the prevalence of noncommunicable comorbidities in people living with HIV [66]. Consistent with this, translocation of bacterial products contributes to persistent immune activation in treated HIV infection, with circulating LPS and sCD14 linked to inflammatory signaling and the progression of subclinical atherosclerosis [60,66]. Collectively, these processes are thought to contribute to the residual inflammatory state that characterizes individuals who have been successfully treated (Figure 1).

## 6. High-Density Lipoproteins and Lipid Metabolism

### 6.1. Protective Functions of HDL Particles

Beyond mediating reverse cholesterol transport, HDL exerts direct anti-inflammatory, antioxidant, and endothelial-protective effects. Foundational epidemiological work by Gordon and colleagues established the inverse relationship between HDL cholesterol concentrations and coronary risk [67]. Rohatgi and colleagues recently cataloged the multiple atheroprotective functions of these particles, including inhibition of LDL oxidation and suppression of endothelial adhesion molecule expression [68]. Contemporary studies in untreated HIV infection show that reduced HDL levels are closely associated with systemic inflammation, with inverse correlations detected between HDL concentrations alongside inflammatory indicators like IL-6 and CRP, indicating that inflammatory processes play a role in the disruption of protective lipid profiles in this population [69,70,71].

### 6.2. HIV-Specific Alterations in HDL Metabolism

Longitudinal MACS data published by Riddler and colleagues demonstrated that seroconversion triggers rapid, sustained reductions in HDL cholesterol [12]. Antiretroviral therapy is frequently associated with increases in total and LDL cholesterol, reflecting partial restoration of lipid metabolism after viral suppression; however, dyslipidemia and altered HDL levels remain common among treated individuals, indicating persistent metabolic perturbations despite effective therapy [72,73,74,75]. Table 5 summarizes characteristic lipid abnormalities associated with infection.

**Figure 2:**
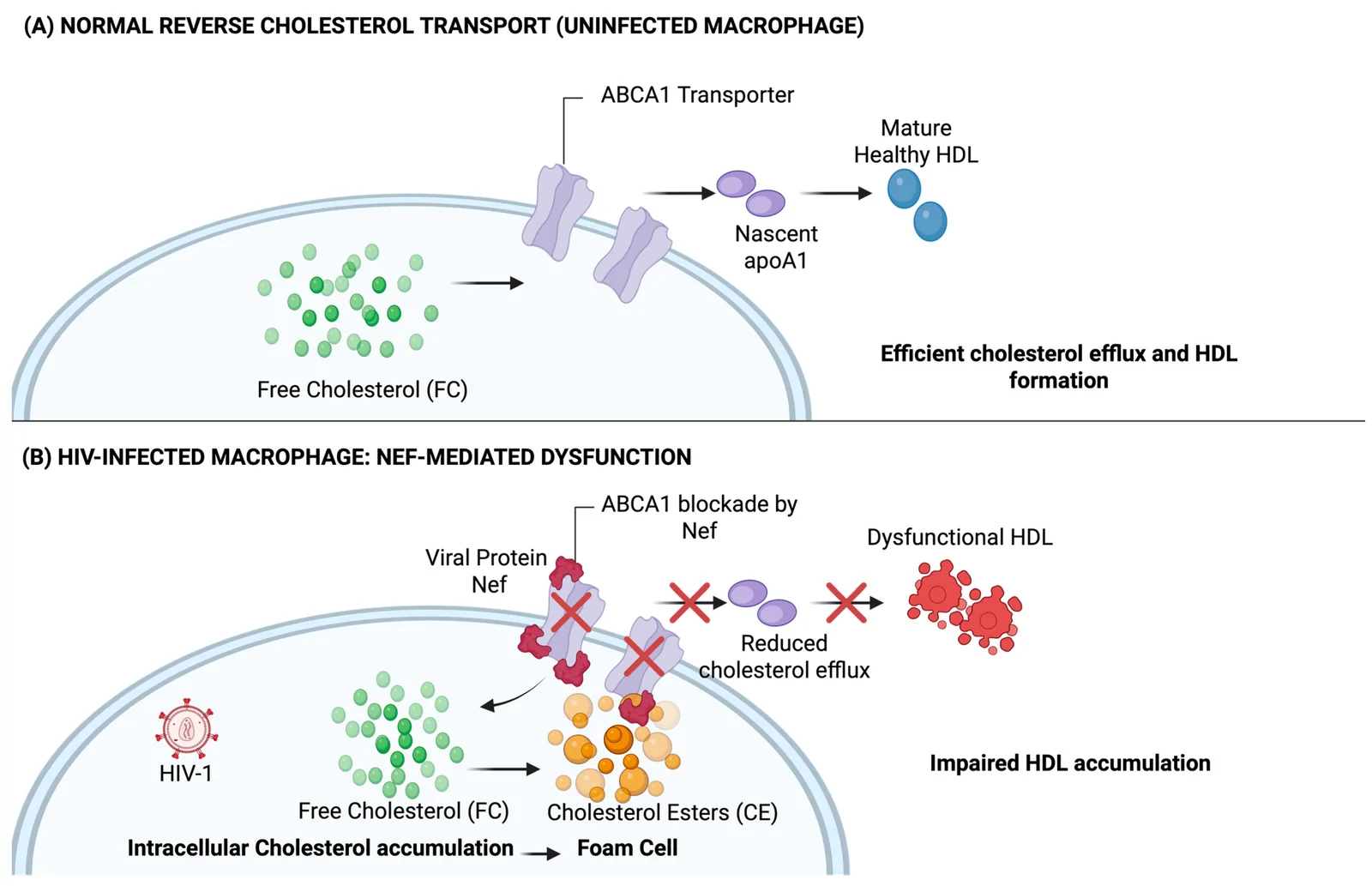
Mechanism of HDL metabolic dysfunction mediated by the HIV Nef protein. Schematic representation of the alteration of reverse cholesterol transport induced by viral infection. (**A**) In macrophages under normal physiological conditions, free cholesterol is efficiently transported to the extracellular space through the ABCA1 transporter, where it is taken up by nascent apoA1 particles to form mature, atheroprotective high-density lipoproteins (HDL). (**B**) In HIV infected macrophages, the viral Nef protein directly interacts with and blocks the intracellular domain of the ABCA1 transporter. This inhibition prevents lipid efflux and leads to two proatherogenic consequences: (1) esterification and massive intracellular accumulation of cholesterol, transforming the macrophage into a foam cell; and (2) defective maturation of nascent apoA1 particles, resulting in the generation of dysfunctional HDL subpopulations (small, dense, and proinflammatory) that lose their protective effects on the vascular endothelium. Created in BioRender. Garibaldi, A. (2026). https://BioRender.com/1bumblt.

A key mechanistic insight into HIV-associated lipid dysregulation was provided by Mujawar and colleagues [76], who demonstrated that the viral accessory protein Nef impairs ATP-binding cassette transporter A1 (ABCA1)-dependent cholesterol efflux from macrophages. By interfering with the principal pathway that transfers excess cellular cholesterol to apolipoprotein A-I, Nef promotes intracellular lipid accumulation and the formation of foam cell–like macrophages, a process linked to atherogenesis. As a consequence of this impaired efflux, lipids accumulate within tissue macrophages, driving their conversion into foam cells (Figure 2). Subsequent lipidomic and functional analyses, summarized by von Eckardstein and colleagues, indicate that HDL particles are structurally and functionally heterogeneous. These studies show that measures of HDL functionality, such as cholesterol efflux capacity and particle composition, more accurately capture HDL’s atheroprotective properties than HDL cholesterol levels alone [78].

## 7. Antiretroviral Therapy and Inflammatory Modulation

### 7.1. Effects of Treatment Initiation

Prompt therapy initiation produces significant increases in HDL particle levels and apolipoprotein A1. Emery and colleagues reported principal clinical endpoints from SMART, definitively establishing continuous therapy as superior to intermittent approaches in mitigating risk [8]. Feinstein and colleagues, in an American Heart Association scientific statement, provided a detailed study of how cardiovascular risk and body composition abnormalities continue despite viral suppression in treated populations [4].

### 7.2. Residual Inflammation Despite Viral Suppression

Despite undeniable treatment benefits, accumulated evidence indicates that systemic inflammation fails to fully normalize. Contemporary biomarker profiling studies show that systemic immune activation persists in people living with HIV despite long-term suppressive antiretroviral therapy (Table 6). For example, longitudinal analyses have shown that markers of inflammation, monocyte activation, and coagulation, such as C-reactive protein, soluble CD14, and D-dimer, decrease after therapy initiation but frequently remain elevated compared with HIV-uninfected individuals [82]. Other clinical studies have similarly reported persistent increases in inflammatory mediators, including interleukin-6, soluble CD163, and platelet activation markers in virologically suppressed patients, particularly in those with subclinical atherosclerosis [83]. Consistent with these observations, cohorts of individuals receiving long-term ART continue to display sustained elevations in monocyte activation markers, such as sCD14, and persistent proinflammatory T-cell responses [84]. Additional investigations have also documented elevated circulating cytokines, including IL-8 and IL-6, in treated individuals, supporting the concept that HIV infection is characterized by a state of residual chronic inflammation despite effective viral suppression [85,86].

**Figure 3:**
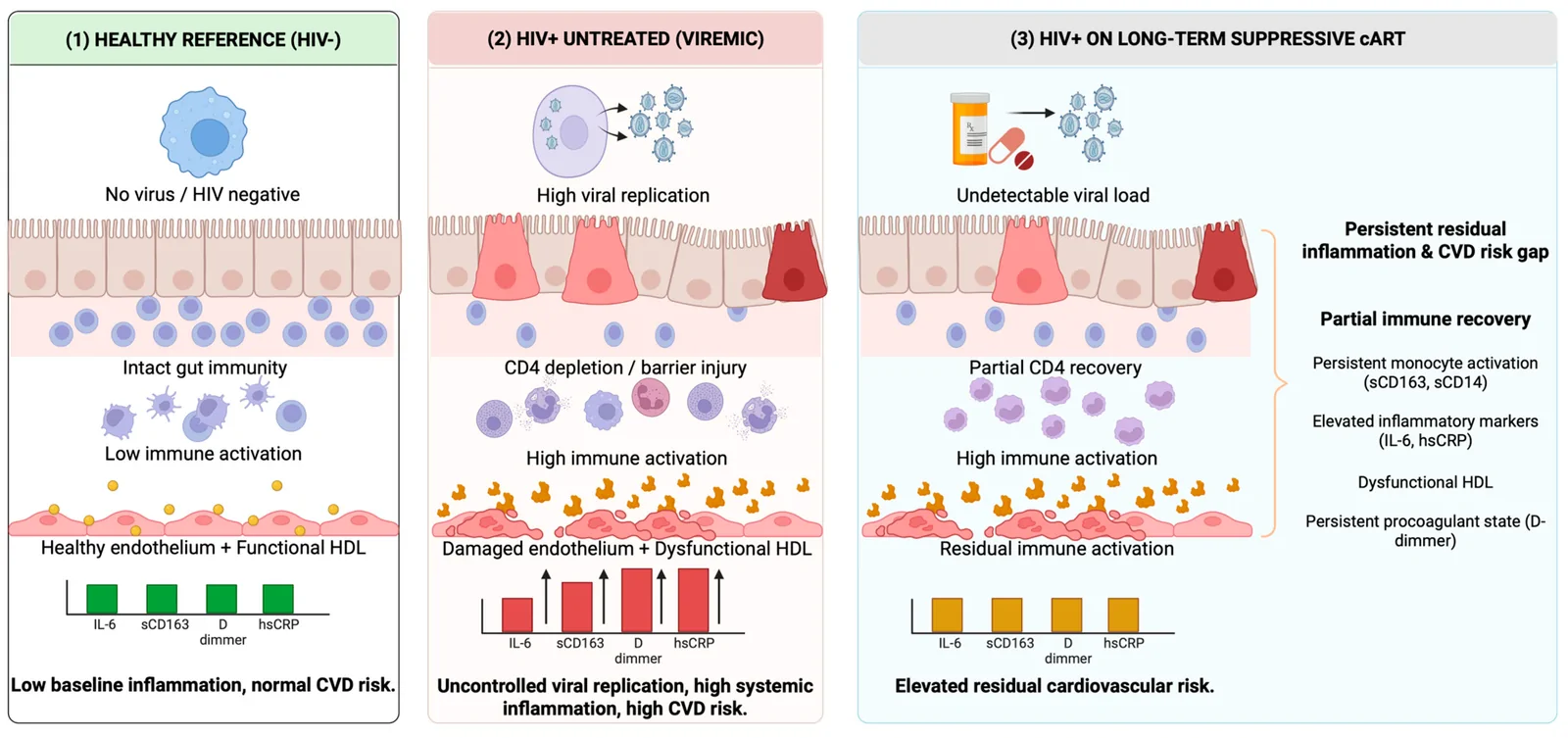
The “inflammatory scar”: persistence of cardiovascular risk despite virological suppression. Schematic representation of the evolution of inflammatory status and cardiovascular risk across several clinical scenarios. (1) Healthy reference (HIV negative): A state of immune homeostasis characterized by low baseline levels of inflammatory biomarkers, functional HDL lipoproteins, intact endothelium, and normal cardiovascular risk. (2) Untreated HIV infection (viremic): Uncontrolled viral replication and massive depletion of CD4+ T lymphocytes trigger severe systemic immune activation. Markedly elevated levels of cytokines (IL-6), markers of monocyte activation (sCD163), and coagulation markers (D-dimer) are observed, together with dysfunctional HDL and endothelial injury, resulting in high cardiovascular risk. (3) HIV infection under long-term antiretroviral therapy (cART): Despite achieving an undetectable viral load and partial immune reconstitution, a “risk gap” persists. Chronic monocyte activation, a procoagulant state, and the dysfunctional HDL phenotype do not fully normalize. This residual systemic inflammation continues to damage the vascular wall, leading to cardiovascular risk that remains significantly higher than in the uninfected population. In the diagrams, black arrows in the upper panels denote viral release (panel 2) and the suppressive action of antiretroviral drugs (panel 3), while vertical upward arrows in the lower bar charts indicate elevated biomarker concentrations relative to the healthy reference. Created in BioRender. Garibaldi, A. (2026). https://BioRender.com/eyeyjw6.

Contemporary cohort studies indicate that people living with HIV develop age-associated comorbidities, including cardiovascular disease, renal impairment, and metabolic conditions, at younger ages than individuals without HIV. Analyses from large clinical cohorts have shown a higher burden of multimorbidity and earlier accumulation of chronic conditions in treated populations compared with uninfected controls [89,90]. Recent reviews additionally show that this pattern reflects the combined effects of chronic immune activation, persistent inflammation, immunosenescence, and long-term exposure to antiretroviral therapy, processes which jointly contribute to accelerated biological aging in HIV infection (Figure 3) [91,92,93].

### 7.3. Differential Effects of Specific Drug Classes

Not all antiretroviral regimens exert equivalent metabolic effects. Recent cohort analyses have shown that switching therapy from tenofovir disoproxil fumarate to tenofovir alafenamide is associated with weight gain and increases in lipid parameters, including total cholesterol, LDL cholesterol, and triglycerides [94,95,96]. These data highlight that contemporary antiretroviral regimens may exert distinct metabolic profiles depending on drug class and formulation. Consistent with these observations, recent reviews emphasize that although modern antiretroviral therapy achieves excellent virological control, long-term treatment may still be accompanied by metabolic complications that warrant careful monitoring and individualized clinical management [97].

## 8. Metabolic, Environmental, and Behavioral Modifiers of Risk

Beyond viral persistence and immune dysregulation, several metabolic, environmental, and behavioral cofactors sustain chronic inflammation and contribute to the elevated cardiovascular risk observed in people living with HIV. Chronic microbial translocation, examined in detail in Section 5.3, is the best characterized of these: the loss of mucosal CD4+ T lymphocytes compromises epithelial barrier integrity and permits systemic leakage of bacterial products such as lipopolysaccharide [65], which activate innate immune signaling through Toll-like receptors, particularly TLR4, driving persistent monocyte activation and increased production of IL-6 and IL-18 even under suppressive therapy [31,60,98]. The factors considered below interact with these pathways and converge on the endothelial and lipid mechanisms already described [18,19,99].

### 8.1. Obesity, Adipose Inflammation, and Metabolic Dysfunction

Obesity and metabolic dysfunction have appeared as important contributors to chronic inflammation and cardiovascular risk in treated HIV populations. As described in Section 7.3, contemporary regimens, particularly integrase strand transfer inhibitors and tenofovir alafenamide, have been associated with weight gain and increased visceral adiposity [94,95,96]. Visceral adipose tissue functions as an active immunometabolic organ that produces inflammatory cytokines, including IL-6, TNF-α, and MCP-1, thereby contributing to systemic low-grade inflammation and endothelial dysfunction [100].

Studies in both HIV-infected and general populations demonstrate that adipose tissue macrophage infiltration and adipokine dysregulation promote insulin resistance, oxidative stress, and vascular inflammation, all of which are key drivers of atherosclerosis [101]. In people living with HIV, these metabolic alterations may interact with persistent immune activation and antiretroviral-associated lipid disturbances, further amplifying cardiovascular risk. Indeed, observational studies have shown that higher body mass index and central adiposity correlate with elevated inflammatory biomarkers, including IL-6 and C-reactive protein, suggesting a mechanistic link between metabolic imbalance and immune activation in this population [102].

### 8.2. Environmental and Lifestyle Determinants

Environmental and lifestyle factors likewise play an important role in affecting cardiovascular risk among people living with HIV. Tobacco smoking remains markedly more prevalent in HIV-infected populations than in the general population and represents one of the strongest modifiable cardiovascular risk factors in this group [103]. Smoking promotes endothelial dysfunction, oxidative stress, and systemic inflammation, thereby synergizing with HIV-related immune activation to accelerate atherogenesis [104].

Additional lifestyle factors such as physical inactivity, dietary patterns, and alcohol consumption may further influence cardiometabolic risk and inflammatory status. Socioeconomic inequalities and barriers to preventive healthcare additionally contribute to the increased burden of cardiovascular disease observed in many HIV-affected populations [19,105]. Together, these environmental and behavioral determinants interact with viral, immunological, and metabolic mechanisms to shape the complex multifactorial landscape of cardiovascular risk in people living with HIV. Differences in antiretroviral regimens, access to lipid-lowering therapy and preventive care across settings carry further implications for how the strategies discussed in this review can be implemented; these are considered in Section 9.

**Figure 4:**
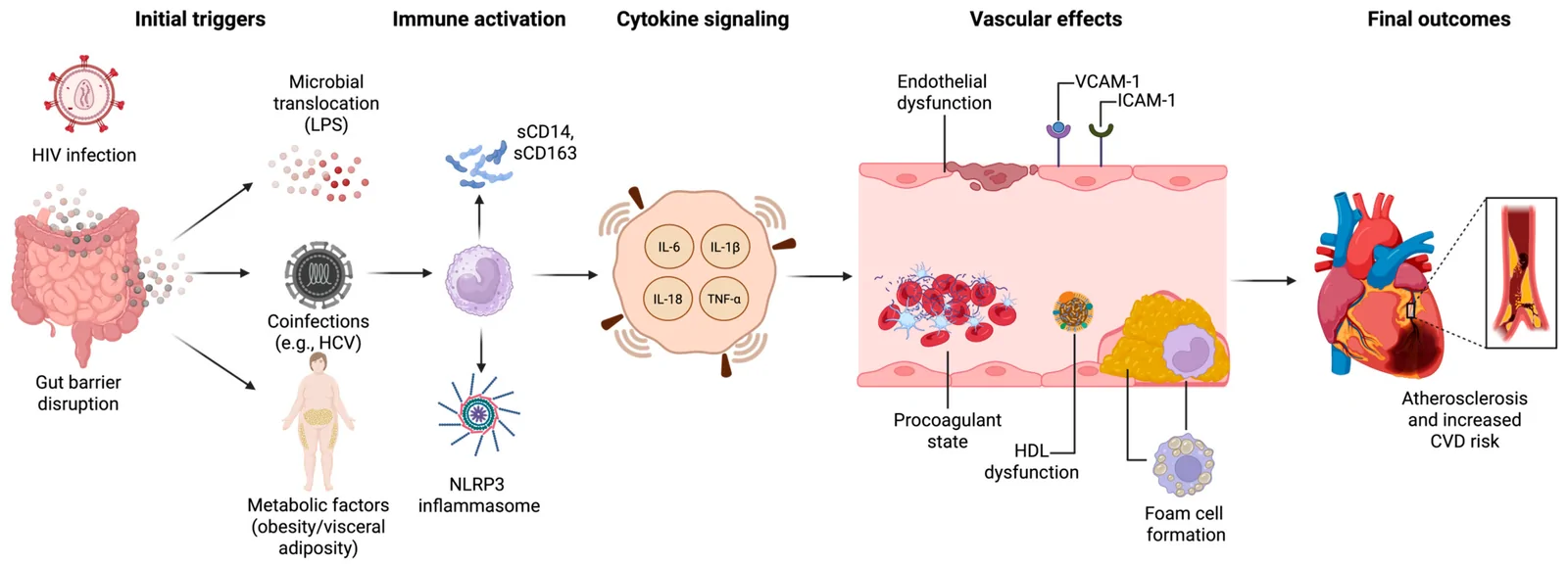
Integrated model of cytokine-mediated cardiovascular risk in HIV infection. HIV infection and associated cofactors, including microbial translocation, coinfections, metabolic disturbances, and daily habits, promote chronic immune activation characterized by monocyte/macrophage stimulation and inflammasome activation. These processes drive the production of proinflammatory cytokines, including IL-6, IL-18, IL-1β, and TNF-α. Cytokine signaling induces endothelial dysfunction, adhesion molecule expression, procoagulant states, platelet activation, HDL dysfunction, and foam cell formation, eventually accelerating atherosclerosis and increasing cardiovascular disease risk.

An integrated overview of the mechanisms discussed throughout this review is summarized in Figure 4.

## 9. Therapeutic Perspectives and Future Directions

The persistence of systemic inflammation despite effective viral suppression has stimulated interest in adjunct treatment interventions. CCR5 antagonists, originally developed as entry inhibitors, have been proposed as dual agents that limit viral replication while modulating the leukocyte recruitment and immune activation implicated in atherosclerosis [41,106,107]. The clinical evidence, however, has not matched this rationale. In a placebo-controlled randomized trial of maraviroc intensification in 45 individuals with incomplete CD4 recovery, immune activation unexpectedly increased rather than decreased: activated CD8+ T-cells rose more in the maraviroc arm than in the placebo arm at week 24 (+2.2% vs. −0.7%; *p* = 0.014), activated CD4+ T-cells declined less (*p* < 0.001), and activation nearly doubled in rectal mucosa [108]. A phase 4 randomized trial in 97 immunological non-responders likewise found no significant advantage in CD4 reconstitution [109]. Evidence on vascular endpoints is more favorable but rests on small pilot studies: in an open-label crossover trial of 21 abacavir-treated men, maraviroc intensification modestly improved flow-mediated dilatation (+0.73% vs. −0.42% during the control period; *p* = 0.049) [110]. CCR5 blockade therefore remains mechanistically attractive but clinically unproven, and adequately powered trials with vascular endpoints would be required before it could be considered as a cardiovascular risk-reduction strategy in this population.

Beyond individual inflammatory markers, integrated multi-omic approaches are being investigated to improve cardiovascular risk prediction in people living with HIV. Mehta and colleagues recently analyzed proteomic and genetic data from participants in the INSIGHT SMART, START, ESPRIT, and FIRST trials, identifying a panel of 14 proteins and 15 genetic variants that discriminated cardiovascular cases from controls better than traditional risk factors alone [111]. The protein score included established markers, such as IL-6 and PLA2G7, as well as novel candidates, including IGFBP7, HGF, and CLEC6A. Notably, functional enrichment analysis showed strong enrichment for inflammation-related pathways, including pathogen-induced cytokine storm signaling and atherosclerosis signaling, with predicted upstream activation of tumor necrosis factor. Combining protein and genetic scores with traditional cardiovascular and HIV-related factors yielded an area under the curve of 0.86, representing a 41% improvement relative to baseline models. Individuals with both scores in the top quartile exhibited nearly 8-fold higher cardiovascular risk compared with others. If verified in independent cohorts, such proteo-genomic risk scores could enable more exact identification of individuals who would benefit most from intensified prevention strategies.

Crucially, the landmark REPRIEVE trial fundamentally shifted the preventive landscape by demonstrating that pitavastatin therapy significantly reduced the incidence of major adverse cardiovascular events in people living with HIV receiving antiretroviral therapy and with low-to-moderate traditional cardiovascular risk. Treatment with pitavastatin was associated with approximately a 35% relative reduction in cardiovascular events compared with placebo (hazard ratio 0.65; 95% CI 0.48–0.90; *p* = 0.002) among 7769 participants; the trial was stopped early for efficacy after a median follow-up of 5.1 years [16]. This practice-changing result has prompted rapid updates to international clinical guidance for cardiovascular prevention in people living with HIV. Following the REPRIEVE findings, the European AIDS Clinical Society now recommends consideration of moderate-intensity statin therapy for primary prevention in individuals with HIV at elevated cardiovascular risk [112].

The translation of these findings into practice will not be uniform across settings. REPRIEVE was conducted at 145 sites in 12 countries and included a majority of non-White participants, which strengthens the generalizability of its result, but implementation depends on conditions that vary widely: availability and cost of pitavastatin, capacity to monitor for muscle-related adverse effects and incident diabetes, and the resources to add a lifelong preventive medication to antiretroviral regimens already competing for adherence. Where pitavastatin is unavailable, whether other statins confer equivalent benefit in this population has not been tested. The antiretroviral landscape differs as well: much of the biomarker evidence summarized in this review derives from cohorts exposed to older regimens, whereas dolutegravir-based therapy is now the global first-line standard, with its own metabolic profile. Cardiovascular risk estimation is a further constraint, since the equations in routine use were derived largely in North American and European populations and perform inconsistently elsewhere. These considerations do not weaken the case for inflammation-informed prevention, but they do mean that the evidence base and the populations bearing most of the global HIV burden are not yet aligned.

Statin therapy does not abolish this risk. In REPRIEVE, the events that continued to occur among treated participants aligned more closely with markers of persistent inflammation than with lipid parameters [17], which is the clearest argument yet for testing interventions aimed at inflammation itself in this population.

Furthermore, targeted anti-inflammatory strategies are gaining attention as complementary approaches to reduce residual cardiovascular risk. The CANTOS trial provided proof of concept that selective inhibition of the interleukin-1β pathway with canakinumab can reduce recurrent cardiovascular events independently of lipid lowering, establishing inflammation as a therapeutic target in atherosclerosis [113]. Building on this paradigm, newer cytokine-directed therapies are under investigation. In the phase 2 RESCUE trial, the IL-6 ligand inhibitor ziltivekimab produced profound reductions in high-sensitivity C-reactive protein and other inflammatory biomarkers associated with atherosclerotic risk [114].

These results must, however, be weighed against the infectious risk inherent to cytokine blockade. In CANTOS, canakinumab was associated with a significantly higher incidence of fatal infection or sepsis than placebo (0.31 vs. 0.18 events per 100 person-years; *p* = 0.02), together with more frequent cellulitis, leukopenia, and thrombocytopenia, although cancer mortality was lower [113]. RESCUE was a phase 2 trial of 264 participants with a biomarker endpoint and was neither designed nor powered to characterize infectious safety [114]. Critically, neither trial enrolled people living with HIV.

This omission matters for the population considered in this review. Residual immune dysfunction, persistent mucosal barrier compromise and a higher burden of latent and opportunistic infection are precisely the features that distinguish treated HIV from the populations studied in CANTOS and RESCUE, and they are the features most likely to modify the risk of sustained IL-1β or IL-6 blockade. The favorable risk–benefit balance observed in HIV-negative participants therefore cannot be assumed to apply here. Dedicated safety and dose-finding studies in people living with HIV, with prespecified infectious endpoints and monitoring of CD4 recovery, are a prerequisite before cytokine-directed therapies can be considered for cardiovascular prevention in this setting.

Parallel observational studies continue to show that persistent immune activation in treated HIV populations correlates with multimorbidity and premature frailty [92]. The need for interventions capable of modulating chronic inflammation in this population is therefore clear; what remains to be established is which of them can be applied safely.

## 10. Limitations

Several limitations shape the interpretation of this review. As outlined in Section 2, the literature was selected to provide an integrative overview rather than a systematic appraisal. Consequently, the emphasis on specific mechanisms reflects the authors’ interpretation of the current evidence landscape.

Additionally, the summarized biomarker values derive from distinct assay platforms and diverse comparator populations with varying age, sex, and risk-factor distributions. Absolute concentrations cannot be directly compared across studies; the magnitude of observed differences must be interpreted strictly within the context of each cohort.

The evidence base also presents significant geographic disparities. Foundational cohorts originate predominantly from North America and Western Europe, leaving populations in sub-Saharan Africa, Asia, and Latin America substantially underrepresented. Regional differences in traditional cardiovascular risk factors, coinfection burden, antiretroviral regimens, and healthcare access inherently limit the global generalizability of these findings.

Most importantly, data linking interleukins to cardiovascular outcomes in HIV remain largely observational. While these associations hold robust prognostic value, they do not establish causality. Current interventional evidence, including CANTOS and REPRIEVE, was not designed to isolate the effects of any single cytokine pathway. Whether the direct modulation of interleukin signaling can independently reduce cardiovascular events in this population requires targeted clinical investigation.

## 11. Conclusions

Evidence from landmark observational studies, complemented by a smaller number of interventional trials, consistently links interleukins and other inflammatory biomarkers to the elevated cardiovascular risk observed in people living with HIV. Biomarkers such as IL-6, markers of monocyte activation (sCD14 and sCD163), and indicators of coagulation activation (D-dimer) remain elevated in this population and are independently associated with subclinical atherosclerosis, major cardiovascular events, and mortality.

The pathophysiological mechanisms underlying this excess cardiovascular risk are multiple and interconnected, including persistent translocation of bacterial products from a compromised intestinal barrier; cytokine-mediated endothelial dysfunction; impaired reverse cholesterol transport, attributed in experimental models to the viral Nef protein; and chronic activation of procoagulant pathways. Although contemporary antiretroviral therapy significantly reduces inflammatory biomarker levels, complete normalization to levels comparable with uninfected controls is generally not achieved, creating an “inflammatory scar” that sustains cardiovascular vulnerability.

Future directions emphasize the incorporation of inflammatory-modulating therapies into routine clinical care. The validation of statin therapy for primary prevention through the REPRIEVE trial is a critical first step. Cytokine-directed therapies represent a plausible next step, but their applicability here remains to be established: the trials that support them were conducted in HIV-negative populations, and the infectious risk of sustained IL-1β or IL-6 blockade has not been characterized in people living with HIV. A better understanding of the relative contribution of each inflammatory pathway will enable the continued development of personalized preventive interventions adapted specifically for people living with HIV.

These conclusions should be read with the nature of the underlying evidence in mind. With the exception of SMART, REPRIEVE, CANTOS, and RESCUE, the data summarized here derive from observational comparisons. The associations between inflammatory biomarkers and cardiovascular outcomes are robust and prognostically informative, but they do not establish that these mediators are causal in humans, and no trial to date has tested whether modulating a single interleukin pathway reduces cardiovascular events in this population.

## Figures and Tables

**Table 1:** Major cohort studies and clinical trials on inflammation and cardiovascular risk in HIV.

Study	Design	Setting and Population	N (HIV-Positive/Uninfected)	Study Period and Follow-Up	Biomarkers or Exposures Evaluated	Main Findings	Refs.
SMART	Randomized controlled trial; nested case–control biomarker analysis	Adults with HIV and a CD4 count above 350 cells/µL randomized to continuous antiretroviral therapy vs. CD4-guided (episodic) therapy; 318 sites in 33 countries	5472 randomized (2720 drug conservation; 2752 viral suppression). Biomarker analysis: 85 deaths and 170 matched controls; 249 drug-conservation and 250 viral-suppression participants for the 1-month biomarker comparison	Randomization from 8 January 2002 to 11 January 2006; mean follow-up 16 months (approximately 3700 person-years per treatment group); enrollment stopped early. Published 2006 and 2008	IL-6, hsCRP, serum amyloid A, serum amyloid P, D-dimer, prothrombin fragment 1 + 2	Interruption of antiretroviral therapy in the CD4-guided arm raised IL-6 and D-dimer; both biomarkers were strongly and independently associated with all-cause mortality.	[7,8,9]
VACS	Observational prospective cohort of people with HIV and matched uninfected veterans; cross-sectional biomarker analysis and prospective mortality analysis	HIV-positive veterans matched with uninfected veterans by age, race or ethnicity, sex, and site; 8 US Department of Veterans Affairs medical centers	1525/843 in the cross-sectional analysis; 1521/829 in the mortality analysis	Specimens collected and banked in 2005–2006; mortality follow-up to 25 July 2013 (median 6.9 years; 414 deaths). Published 2012 and 2016	IL-6, D-dimer, sCD14	Median IL-6 is higher in HIV-positive than in uninfected veterans, but the adjusted prevalence of elevated (above the 75th percentile) IL-6, D-dimer and sCD14 does not differ overall and emerges only after stratification by viraemia or CD4 count. These biomarkers independently predict all-cause mortality and partially account for the excess mortality of HIV-positive vs. uninfected people.	[10,11]
MACS	Prospective cohort; analysis of stored sera from seroconverters who later initiated antiretroviral therapy	HIV seroconverters within a prospective cohort of men who have sex with men; 4 US centers (Pittsburgh, Baltimore, Chicago, Los Angeles)	50 of 517 HIV seroconverters analyzed	Stored serum samples from 1984 to 2002; 6 time points over an average of 12 years. Published 2003	Total cholesterol, LDL-C, HDL-C, triglycerides	Total cholesterol, LDL-C and HDL-C decline after HIV seroconversion; with antiretroviral therapy, total cholesterol and LDL-C rise, but HDL-C does not return to pre-seroconversion levels.	[12]
WIHS/MACS	Prospective cohort with repeated B-mode carotid artery ultrasound	Women (WIHS) and men (MACS) with and without HIV; US sites	778 women (74% HIV-positive) and 503 men (65% HIV-positive)	Imaging between 2004 and 2013; carotid plaque formation assessed over 7 years. Published 2017	sCD163, sCD14, galectin-3, galectin-3 binding protein	Higher sCD14 and sCD163 are associated with carotid plaque formation (focal intima-media thickness above 1.5 mm) over 7 years of follow-up.	[13]
D:A:D	Prospective observational multicohort collaboration (11 contributing cohorts)	People with HIV in routine clinical care; 188 clinics in 21 countries in Europe, the United States and Australia	23,468 in the 2003 analysis; 33,347 in the 2008 analysis	Enrollment from December 1999 to April 2001, with follow-up to February 2002; 36,199 person-years and 126 myocardial infarctions in the 2003 analysis, and 157,912 person-years and 517 myocardial infarctions in the 2008 analysis. Published 2003 and 2008	Cumulative and recent antiretroviral exposure; incident myocardial infarction	Specific antiretroviral exposures, such as abacavir, didanosine, and older protease inhibitors, are associated with an increased risk of myocardial infarction and other major cardiovascular events.	[14,15]
REPRIEVE	Phase 3, randomized, double-blind, placebo-controlled trial	Adults with HIV receiving antiretroviral therapy with a low-to-moderate traditional cardiovascular risk; 145 sites in 12 countries	7769 enrolled (3888 pitavastatin; 3881 placebo) of 10,865 screened; 31.1% women and 65.2% non-White	Enrollment from 26 March 2015 to 31 July 2019; stopped early for efficacy after a median follow-up of 5.1 years (interquartile range 4.3–5.9). Published 2023	Pitavastatin calcium 4 mg daily vs. placebo; major adverse cardiovascular events	Major adverse cardiovascular events occurred at 4.81 per 1000 person-years with pitavastatin vs. 7.32 per 1000 person-years with placebo (hazard ratio 0.65; 95% CI 0.48–0.90; ***p*** = 0.002), establishing statin therapy for primary cardiovascular prevention in this population. In a secondary biomarker analysis of 7005 participants followed for a median of 5.6 years, IL-6 and high-sensitivity troponin showed the strongest associations with residual MACE, with population attributable fractions of 18.6% and 17.2% for IL-6 and hsCRP, respectively.	[16,17]

CD4, cluster of differentiation 4; CI, confidence interval; D:A:D, Data Collection on Adverse Events of Anti-HIV Drugs; HDL-C, high-density lipoprotein cholesterol; HIV, human immunodeficiency virus; hsCRP, high-sensitivity C-reactive protein; IL-6, interleukin-6; LDL-C, low-density lipoprotein cholesterol; MACE, major adverse cardiovascular events; MACS, Multicenter AIDS Cohort Study; REPRIEVE, Randomized Trial to Prevent Vascular Events in HIV; sCD14, soluble CD14; sCD163, soluble CD163; SMART, Strategies for Management of Antiretroviral Therapy; US, United States; VACS, Veterans Aging Cohort Study; WIHS, Women’s Interagency HIV Study. The WIHS row reports a pooled WIHS and MACS analysis. Study designs, comparator populations and assay platforms differ between studies, so the values reported are not directly comparable across rows. Ref.: References.

**Table 2:** Cross-sectional concentrations of inflammation, coagulation, monocyte/endothelial-activation, and renal biomarkers in antiretroviral-treated HIV vs. HIV-negative controls.

Biomarker (Units)	HIV—Median (Dispersion)	HIV-Negative Control—Median (Dispersion)	*p*	Study Population (Comparison)	Ref.
IL-6 (pg/mL)	2.64 (1.55–4.14)	1.23 (0.79–1.92)	<0.001	SMART (ART, HIV-RNA ≤ 400) vs. MESA, 45–76 years	[26]
hsCRP (µg/mL) ^a^	2.83 (1.07–6.80)	2.17 (0.94–4.70)	<0.001	SMART (ART, HIV-RNA ≤ 400) vs. MESA, 45–76 years	[26]
D-dimer (µg/mL)	0.29 (0.17–0.57)	0.20 (0.13–0.35)	<0.001	SMART (ART, HIV-RNA ≤ 400) vs. MESA, 45–76 years	[26]
Cystatin C (mg/L)	1.00 (0.86–1.16)	0.85 (0.76–0.96)	<0.001	SMART (ART, HIV-RNA ≤ 400) vs. MESA, 45–76 years	[26]
sCD163 (ng/mL)	792 (562–1025)	448 (362–526)	<0.001	Case–control, 26 HIV after 48-wk ART vs. 23 controls	[27]
sTWEAK (pg/mL)	325 (299–386)	468 (410–512)	<0.001	Case–control, 26 HIV after 48-wk ART vs. 23 controls	[27]
Fibrinogen, men (mg/dL) ^b^	345 (95% CI 337–356)	320 (311–337)	<0.0001 (adjusted)	FRAM vs. CARDIA controls, men 33–45 years	[28]
sVCAM-1 (ng/mL) ^c^	659 (239–17,145)	454 (66–1808)	0.002	Cross-sectional, 73 HIV on ART ≥24 wk vs. 21 controls	[29]

Values are median (interquartile range) unless otherwise noted; *p* for HIV vs. HIV-negative control. Comparator populations, assay platforms and inclusion criteria differ between rows, so values are not comparable across rows. ^a^ hsCRP in µg/mL is numerically equivalent to mg/L; the source reports µg/mL. ^b^ Median with 95% CI (not IQR). In women the difference was not significant (373, 95% CI 347–383, vs. 352, 95% CI 330–367; *p* = 0.79). ^c^ Median with range (not IQR); 73 HIV-positive patients and 21 controls. In the separate case–control study of ref. [27], sVCAM-1 was likewise elevated in HIV and was not modified by 48 weeks of antiretroviral therapy. Ref.: References.

**Table 3:** Odds of elevated (>75th percentile) inflammation, coagulation, and monocyte-activation biomarkers by HIV virological/immunological status in the VACS Biomarker Cohort (1525 HIV-positive; 843 HIV-negative veterans).

Biomarker	Comparison (vs. Uninfected Veterans)	OR (95% CI) for Elevated Biomarker (>75th pct)	Significance	Ref.
IL-6	Overall HIV	1.18 (0.96–1.44)	NS	[11]
IL-6	HIV, HIV-RNA ≥ 500 copies/mL	1.54 (1.14–2.09)	Significant	[11]
IL-6	HIV, CD4 < 200 cells/µL	2.25 (1.60–3.16)	Significant	[11]
D-dimer	Overall HIV	0.91 (0.74–1.11)	NS	[11]
D-dimer	HIV, HIV-RNA ≥ 500 copies/mL	1.97 (1.44–2.71)	Significant	[11]
D-dimer	HIV, CD4 < 200 cells/µL	1.68 (1.22–2.32)	Significant	[11]
sCD14	Overall HIV	1.16 (0.95–1.42)	NS	[11]
sCD14	HIV, CD4 < 200 cells/µL	2.60 (1.64–4.14)	Significant	[11]

Odds ratios in rows labeled “Overall HIV” are adjusted for age and race/ethnicity; rows stratified by HIV-1 RNA or CD4 count are additionally adjusted for comorbid conditions. The reference group is HIV-uninfected veterans, and “elevated” denotes a value above the 75th percentile. Median IL-6 was higher in HIV-positive than in uninfected veterans (2.08 vs. 1.79 pg/mL; *p* < 0.01). NS, not significant. Ref.: References.

**Table 4:** Mechanisms linking the main cytokines and chemokines to atherogenesis in HIV infection.

Type of Evidence	Cytokine	Proatherogenic Mechanisms	Cardiovascular Consequences	Refs.
Narrative reviews (general vascular biology); HIV-specific evidence in [39].	IL-6	Hepatic induction of acute-phase reactants (C-reactive protein, fibrinogen, serum amyloid A); endothelial activation through gp130 signaling, with adhesion-molecule expression and monocyte recruitment; contribution to macrophage maturation and activation within the lesion.	Accelerated atherosclerosis, systemic endothelial dysfunction, and a prothrombotic state; in people with HIV, association with greater carotid intima-media thickness and with major adverse cardiovascular events.	[36,37,39]
Narrative review [39]; in vitro and in situ study in human atheroma, non-HIV [40].	IL-18	Induction of IFN-γ in vascular smooth muscle cells and macrophages, strongly potentiated in synergy with IL-12; functional IL-18Rα/β receptor on endothelial cells, vascular smooth muscle cells, and plaque macrophages; polarization of the response toward Th1, with secondary induction of IL-6, IL-8, and ICAM-1.	Amplification of the Th1/IFN-γ response; activation of macrophages and vascular smooth muscle cells; thinning of the fibrous cap and plaque instability.	[39,40]
Narrative reviews [39,41]; murine model of cholesterol-crystal atherogenesis, non-HIV [42].	IL-1β	Activation of the NLRP3 inflammasome by cholesterol crystals, with caspase-1-dependent maturation of IL-1β (and IL-18) and pyroptosis, a step required for atherogenesis; in people with HIV, NLRP3 inflammasome activation in macrophages with foam-cell formation.	Sustained vascular inflammation, leukocyte recruitment, and progression and instability of the atherosclerotic plaque.	[39,41,42]
Narrative reviews; HIV-specific evidence in [39].	TNF-α	Activation of NF-κB, with endothelial adhesion-molecule expression and endothelial dysfunction and apoptosis; induction of insulin resistance and lipolysis (activation of hormone-sensitive lipase and adipose triglyceride lipase), with a proatherogenic lipid profile; in people with HIV, T-cell-derived TNF-α promotes monocyte procoagulant activity within atherosclerotic plaque.	Endothelial dysfunction and atherogenesis; dyslipidemia and insulin resistance (features of the metabolic syndrome); a prothrombotic state.	[39,43]
Narrative review [44]; MCP-1–deficient murine model, non-HIV [45].	MCP-1/CCL2	Chemoattraction of circulating monocytes into the subendothelial space through the CCL2-CCR2 axis, with differentiation into macrophages and foam-cell formation; deletion of CCL2 or CCR2 reduces atherosclerotic lesion burden in murine models.	Progression of the atherosclerotic lesion, subendothelial lipid accumulation, and expansion of the necrotic core.	[44,45]
Narrative reviews [39,41]	IL-10	Anti-inflammatory cytokine that deactivates macrophages and T lymphocytes and inhibits NF-κB and proinflammatory cytokine synthesis; its deficiency increases atherosclerotic burden, with greater T-cell infiltration and IFN-γ expression; in people with HIV, IL-10 is associated with lower plaque burden, with possible plasma-tissue discordance.	Net atheroprotective effect; its relative deficiency favors a proinflammatory imbalance.	[39,41]

Abbreviations: CCR2, C-C chemokine receptor type 2; HIV, human immunodeficiency virus; ICAM-1, intercellular adhesion molecule-1; IFN-γ, interferon gamma; IL, interleukin; MCP-1/CCL2, monocyte chemoattractant protein-1/C-C motif chemokine ligand 2; NF-κB, nuclear factor kappa B; NLRP3, NOD-, LRR- and pyrin domain-containing protein 3; Th1, T helper type 1; TNF-α, tumor necrosis factor alpha. Ref.: References. The references supporting this table correspond to narrative reviews, experimental models and mechanistic studies rather than to named cohorts or trials; the type of evidence is therefore specified in place of a study name. Mechanisms marked as non-HIV derive from general vascular biology or experimental models; their relevance to HIV-associated atherosclerosis is inferred rather than directly demonstrated in people living with HIV.

**Table 5:** Alterations within high-density lipoprotein metabolism associated with HIV infection.

Study or Evidence Source	Lipid Parameter	Effect of HIV Infection	Pathophysiological Mechanism	Refs.
MACS [12]; Marín-Palma et al., cross-sectional study [69]; Mujawar et al., in vitro macrophage model [76].	Total HDL cholesterol	Marked decline after seroconversion (on the order of 12 mg/dL); remains low and does not return to pre-infection levels with ART.	Nef-mediated impairment of ABCA1-dependent cholesterol efflux; HDL lowering driven by chronic inflammation.	[12,69,76]
Mujawar et al., in vitro macrophage model [76]; INSIGHT SMART lipoprotein substudy [77].	Total HDL particles (HDL-P)	Lower HDL-P is associated with cardiovascular events in people with HIV, an association that persists after individual adjustment for hsCRP, IL-6 or D-dimer and is attenuated only with simultaneous adjustment for all three; treatment interruption lowers total HDL-P.	Impaired reverse cholesterol transport; inflammation-driven HDL remodeling.	[76,77]
INSIGHT SMART [77]; von Eckardstein et al., narrative review [78].	Small HDL particles (small HDL-P)	Reduced; lower small HDL-P is independently associated with cardiovascular events in the SMART study.	Loss of the anti-inflammatory and antioxidant functions carried by HDL particles.	[77,78]
Mujawar et al., in vitro macrophage model [76].	Cholesterol efflux capacity	Reduced in people with HIV	ABCA1 downregulation with cholesterol accumulation in macrophages and foam-cell formation.	[76]
AMORIS [79]; Swiss HIV Cohort Study, nested case–control [80].	ApoB/ApoA-I ratio	Increased; small dense LDL and ApoB are associated with coronary events in people with HIV on antiretroviral therapy [80], while the predictive superiority of the ApoB/ApoA-I ratio over conventional lipid indices derives from general-population data [79].	Imbalance between atherogenic (ApoB) and protective (ApoA-I) lipoproteins.	[79,80]
Markakis et al., narrative review [81].	HDL composition	Shift toward a proinflammatory, dysfunctional HDL: enrichment in serum amyloid A and reduction or oxidation of ApoA-I.	Inflammation-driven remodeling of the HDL proteome and lipidome; dysfunctional HDL promotes monocyte activation.	[81]

Abbreviations: ABCA1, ATP-binding cassette transporter A1; AMORIS, Apolipoprotein-related Mortality Risk study; ApoA-I, apolipoprotein A-I; ApoB, apolipoprotein B; ART, antiretroviral therapy; HDL, high-density lipoprotein; HDL-P, HDL particle number; HIV, human immunodeficiency virus; hsCRP, high-sensitivity C-reactive protein; IL-6, interleukin-6; LDL, low-density lipoprotein; MACS, Multicenter AIDS Cohort Study; SMART, Strategies for Management of Antiretroviral Therapy. Ref.: References.

**Table 6:** Effects of antiretroviral therapy on inflammatory and metabolic biomarkers.

Biomarker	Study or Evidence Source	Effect of ART Initiation	Status During Sustained Suppressive ART	Returns to Levels of Uninfected Controls	Refs.
IL-6	INSIGHT SMART vs. MESA and CARDIA [26]; Illanes-Álvarez et al., cross-sectional study with matched controls [83]; INSIGHT SMART, ESPRIT and SILCAAT, pooled prognostic analysis [87].	Reduced with viral suppression but not normalized	Remains elevated in treated individuals with suppressed viremia and predicts serious non-AIDS events and death	No	[26,83,87]
hsCRP	INSIGHT SMART vs. MESA and CARDIA [26]; RV254/SEARCH acute HIV cohorts, Thailand [82].	Decrease after therapy	Frequently remains higher than in HIV-negative controls	No	[26,82]
sCD163	Beltrán et al., pre-post ART study with healthy controls [27]; Burdo et al., cross-sectional coronary CT angiography study [88].	Decrease with ART	Remains elevated despite viral suppression and is independently associated with noncalcified coronary plaque	No	[27,88]
sCD14	SMART, nested case–control [31]; RV254/SEARCH acute HIV cohorts [82]; Watanabe et al., age-matched controlled study in older adults [84].	Decreases with treatment but remains above control levels	Remains elevated during long-term suppressive ART	No	[31,82,84]
HDL-C	MACS [12]; Lembas et al., 1-year prospective cohort [72].	Modest increase after ART initiation (regimen-dependent)	Does not return to pre-seroconversion levels	Not assessed	[12,72]

Abbreviations: AIDS, acquired immunodeficiency syndrome; ART, antiretroviral therapy; CARDIA, Coronary Artery Risk Development in Young Adults; CT, computed tomography; ESPRIT, Evaluation of Subcutaneous Proleukin in a Randomized International Trial; HDL-C, high-density lipoprotein cholesterol; HIV, human immunodeficiency virus; hsCRP, high-sensitivity C-reactive protein; IL-6, interleukin-6; INSIGHT, International Network for Strategic Initiatives in Global HIV Trials; MACS, Multicenter AIDS Cohort Study; MESA, Multi-Ethnic Study of Atherosclerosis; sCD14, soluble CD14; sCD163, soluble CD163; SEARCH, South East Asia Research Collaboration in HIV; SILCAAT, Subcutaneous Interleukin-2 in Patients with Low CD4 Counts under Active Antiretroviral Therapy; SMART, Strategies for Management of Antiretroviral Therapy. Ref.: References.

## Data Availability

No new data were created or analyzed in this study. Data sharing is not applicable to this article.
